# Added Value of HER-2 Amplification Testing by Multiplex Ligation-Dependent Probe Amplification in Invasive Breast Cancer

**DOI:** 10.1371/journal.pone.0082018

**Published:** 2013-12-04

**Authors:** Chantal C. H. J. Kuijpers, Cathy B. Moelans, Henk-Jan van Slooten, Anja Horstman, John W. J. Hinrichs, Shaimaa Al-Janabi, Paul J. van Diest, Mehdi Jiwa

**Affiliations:** 1 Symbiant Pathology Expert Centre, Alkmaar, The Netherlands; 2 University Medical Centre Utrecht, Department of Pathology, Utrecht, The Netherlands; University of Navarra, Spain

## Abstract

**Background:**

HER-2 is a prognostic and predictive marker, but as yet no technique is perfectly able to identify patients likely to benefit from HER-2 targeted therapies. We aimed to prospectively assess the added value of first-line co-testing by IHC, and multiplex ligation-dependent probe amplification (MLPA) and chromogenic *in situ* hybridization (CISH).

**Methods:**

As local validation, HER-2 MLPA and CISH were compared in 99 breast cancers. Next, we reviewed 937 invasive breast cancers, from 4 Dutch pathology laboratories, that were prospectively assessed for HER-2 by IHC and MLPA (and CISH in selected cases).

**Results:**

The validation study demonstrated 100% concordance between CISH and MLPA, if both methods were assessable and conclusive (81.8% of cases). Significant variation regarding percentages IHC 0/1+ and 2+ cases was observed between the laboratories (p<0.0001). Overall concordance between IHC and MLPA/CISH was 98.1% (575/586) (Kappa = 0.94). Of the IHC 3+ cases, 6.7% failed to reveal gene amplification, whereas 0.8% of the IHC 0/1+ cases demonstrated gene amplification. Results remained discordant after retrospective review in 3/11 discordant cases. In the remaining 8 cases the original IHC score was incorrect or adapted after repeated IHC staining.

**Conclusions:**

MLPA is a low-cost and quantitative high-throughput technique with near perfect concordance with CISH. The use of MLPA in routinely co-testing all breast cancers may reduce HER-2 testing variation between laboratories, may serve as quality control for IHC, will reveal IHC 0/1+ patients with gene amplification, likely responsive to trastuzumab, and identify IHC 3+ cases without gene amplification that may respond less well.

## Introduction

In breast cancer patients, the HER-2 oncogene is both a prognostic and a predictive marker, but as yet no technique is perfectly able to identify patients likely to benefit from HER-2 targeted therapies. The HER-2 oncogene is amplified and/or overexpressed in approximately 10-15% of human breast cancers [1]. Overexpression of the HER-2 oncogene is associated with poor prognosis and resistance to chemotherapy and hormonal therapy [2]. More importantly, HER-2 status identifies patients likely to benefit from treatment with the recombinant humanized monoclonal antibody trastuzumab and the small molecule tyrosine kinase inhibitor lapatinib [3]; [4]. As both these therapies are expensive, and trastuzumab is associated with serious, particularly cardiotoxic, side-effects, the ASCO/CAP guidelines [5] stipulate that trastuzumab therapy is only applicable for patients who strongly overexpress the HER-2 protein (3+) and those who present with equivocal HER-2 protein levels (2+) with confirmed gene amplification.

Accurate HER-2 assessment is required, and an accurate, robust and reproducible assay is essential. The most commonly used method to assess HER-2 status is immunohistochemistry (IHC), probably because it is widely available and relatively inexpensive. However, despite of a standardized testing protocol, IHC has proven to be sensitive to pre-analytical variation and inter- and intra-observer variability [6]–[8].

Assessment of HER-2 gene amplification status, usually limited to the IHC equivocal cases, is most commonly performed by fluorescence (FISH) and chromogenic *in situ* hybridization (CISH). These two techniques, demonstrating excellent concordance [9], are less sensitive to pre-analytical variation than IHC, but they are labor-intensive, expensive and somewhat difficult to interpret.

A cost-effective, easy-to-perform and quantitative high-throughput technique for routinely assessing HER-2 gene amplification status may be the PCR-based multiplex ligation-dependent probe amplification (MLPA) technique. Moerland *et al* [10] first described MLPA for the use of HER-2 gene amplification detection in 2006. In their study and in other studies, MLPA has proven to be a reliable, less expensive and high-throughput alternative, highly concordant with ISH [11]–[14].

HER-2 protein expression and gene amplification are not in complete accordance. Previous studies have demonstrated IHC 0/1+ breast cancers with HER-2 gene amplification as well as IHC 3+ cases without gene amplification [7]; [10]–[12]; [15]–[19]. Therefore, in April 2011 we at Symbiant B.V. started co-testing for HER-2 with IHC and MLPA in every invasive breast cancer case, instead of only the equivocal cases. In the University Medical Centre (UMC) Utrecht co-testing of every invasive breast cancer case was first applied in 2004.

The aim of our study was to prospectively assess the added value of first-line co-testing for HER-2 using a combination of IHC and MLPA in routine pathology practice.

## Materials and Methods

### Ethics statement

Since we used archival pathology material which does not interfere with patient care and does not involve the physical involvement of the patient, no ethical approval is required according to Dutch legislation [the Medical Research Involving Human Subjects Act (Wet medisch-wetenschappelijk onderzoek met mensen, WMO [20])]. Use of anonymous or coded left over material for scientific purposes is part of the standard treatment contract with patients, and therefore informed consent procedure was not required according to our institutional medical ethical review board. This has also been described by van Diest *et al* [21]. We assume that our study is subjected to exemption from the Federal regulation as has been suggested below: Exemption 4 includes research involving the collection or study of existing data, documents, records, pathological specimens, or diagnostic specimens, if these sources are publicly available or if the information is recorded by the investigator in such a manner that subjects cannot be identified, directly or through identifiers linked to the subjects [22]. Also, this is based on the Dutch guidelines for research [23].

### MLPA validation study

We conducted a validation study prior to implementing MLPA for assessing HER-2 amplification status in invasive breast cancer into the diagnostics of the 3 Symbiant laboratories (Alkmaar Medical Centre, Zaandam Medical Centre and Westfriesgasthuis Hoorn, The Netherlands) in April 2011. We triple-tested 99 invasive breast cancer cases by IHC, MLPA and CISH. The aim of this validation study was to locally determine the concordance rates of these techniques.

### Patient cohorts

**Figure 1:**
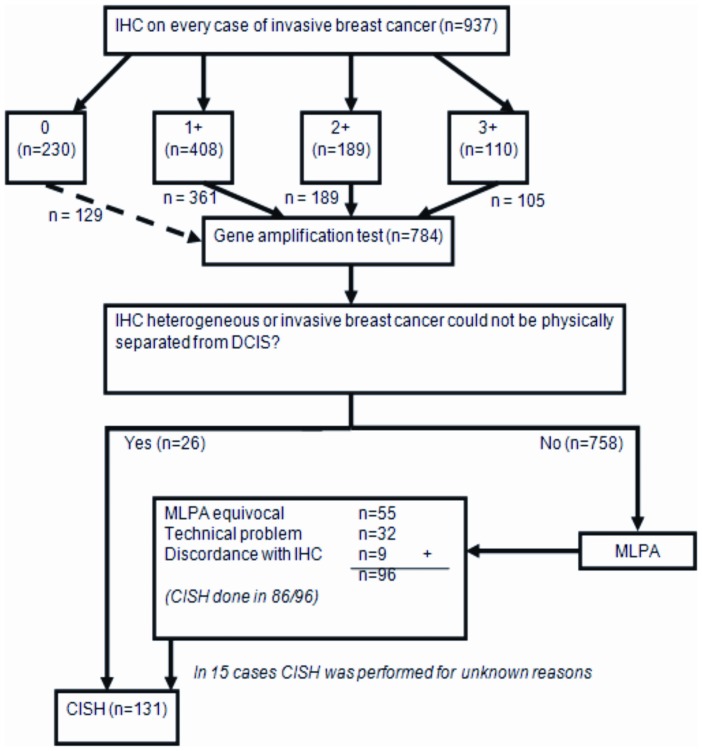
HER-2 co-testing protocol. IHC was performed on every case of invasive breast cancer. 129/230 cases with IHC score 0, 361/409 IHC 1+ cases, all 189 IHC 2+ cases and 105/110 IHC 3+ cases were tested by either MLPA, CISH or both. MLPA was performed in 758 cases and reflex CISH was performed in 86/96 cases with an equivocal MLPA result, a technical problem or a discordant result from IHC. The remaining 10/96 cases did not undergo reflex CISH either due to an insufficient amount of tumor tissue or an unknown reason. Furthermore, in 16 cases, CISH was performed beyond the protocol. Finally, CISH was performed instead of MLPA in 26 cases (4 were immunohistochemically heterogeneous, in 4 cases invasive tumor could not be physically separated from DCIS, in 12 cases tumor cell percentage was low, in 3 cases no MLPA was requested accidentally and in 3 cases the reason why no MLPA was performed could not be elucidated).

We reviewed the pathology reports of consecutive cohorts of 920 invasive breast cancer patients from 4 laboratories in The Netherlands (figure 1). For 720 patients, their HER-2 status was determined in one of the 3 laboratories of Symbiant B.V. between April 2011 and February 2012. For the other 200 patients, their HER-2 status was determined in the pathology laboratory of the UMC Utrecht between January 2010 and December 2011. Eighteen patients had multiple tumors that were included as separate cases. We excluded 2 cases which had only a very small micro-invasive component. In total 937 tumors were thereby analyzed in this study. Of these cases, 78.7% were resections, 19.9% were biopsies, and 3.6% were metastases.

### Immunohistochemistry

IHC staining was performed fully automated using the Bond-III or the Bond-Max™ immunostainers (Leica, Wetzlar, Germany), according to the manufacturers’ instructions on 4 µm thick tissue paraffin sections. In addition, separate paraffin sections with 4 human breast cancer cell lines (0, 1+, 2+ and 3+ intensity, provided by Leica), were taken along as controls to validate staining runs. Moreover, a small control tissue array containing a 0, 1+ and 3+ breast tumor sample was mounted on the same slide as the tissue section of the tumor sample to be analyzed to serve as negative and positive controls.

Antigen retrieval was performed for 20 minutes with EDTA (100 °C), and slides were incubated for 25 minutes with HER-2 rabbit monoclonal antibody (clone SP3, Neomarkers, Fremont, CA, USA; dilution 1:50). Peroxidase blocking was performed during 5 minutes, and slides were subsequently incubated with poly-HRP (ready-to-use) for 8 minutes, with DAB for 10 minutes and counterstained with hematoxylin for 5 minutes to visualize cell nuclei.

HER-2 protein expression was scored by consensus of 2 observers as negative (0/1+), equivocal (2+) or positive (3+), according to the 2007 ASCO/CAP guidelines [5]. First, either a general or a breast pathologist assessed and scored the staining in his/her daily routine. Thereafter, every invasive breast cancer case was reviewed by a breast pathologist before being discussed at the multidisciplinary meeting. In case of a discrepancy, a second breast pathologist was consulted, resulting in a definite consensus score. Samples that were originally reported to comprise a range of scores (i.e. 1-2+) were reviewed for the sake of this study by a breast pathologist (HS) in order to provide an unambiguous score. DCIS areas were excluded from the evaluation, and cytoplasmic staining was ignored.

### Multiplex Ligation-Dependent Probe Amplification (MLPA)

Invasive tumor areas as identified on serial H&E sections were harvested from 1–2 whole 4 µm thick paraffin sections (corresponding to approximately 1–2 square cm tumor tissue) using a scalpel as before [19]; [24]. Paraffin sections containing normal breast and blood (UMC Utrecht) and normal lymph nodes (Symbiant B.V.) were taken along for normalization. DNA was isolated from these tissue fragments by a direct lysis method with proteinase K (Roche, Almere, The Netherlands). After centrifugation, DNA was used in the MLPA analysis, according the manufacturers’ instructions, using the P078-B1 probemix (MRC Holland, Amsterdam, The Netherlands) [25]. All tests were performed in duplicate in an ABI 9700 PCR machine (Applied Biosystems, Foster City, CA, USA) or a Tprofessional thermocycler (Biometra, Goettingen, Germany). The PCR products were analyzed on an ABI3730 or ABI310 capillary sequencer (Applied Biosystems). Normal breast tissue was used as a control.

Gene copy numbers were analyzed using Genemapper (Applied Biosystems) and Coffalyser (version 7.0) software (MRC-Holland). To confirm validity of the HER-2 amplification results, copy number ratio results of 11 internal reference probes, included in the P078-B1 probemix, were checked. If ≥ 2 reference probes were aberrant, test results were considered invalid or inconclusive.

Copy number ratio for every probe (including HER-2 probes) was obtained by dividing the relative peak height for each probe in the tumor tissue by the relative value of the same peak for the reference DNA samples. To make the normalization robust the algorithm makes use of every MLPA probe signal, set as a reference probe for normalization to produce an independent relative ratio. All ratios were finally normalized by setting the median of the tumor to reference DNA copy number ratios of the reference probes in the probe mixture to 1.0 [26].

The mean of all 4 HER-2 probe copy number peaks in duplicate was calculated. If the MLPA copy number ratio was < 1.3 HER-2 status was defined as normal, a value between 1.3 and 2.0 as equivocal and values > 2.0 as amplified [27].

### Chromogenic *in situ* hybridization (CISH)

CISH was performed in case of technical problems with MLPA (aberrant copy numbers of reference probes, unreliable duplicates, poor quality of the DNA) or if the MLPA result was equivocal (MLPA value between 1.3 and 2.0) or discordant with the IHC score. In these cases, the CISH result determined the definite amplification status.

Moreover, because MLPA is a non-morphologic technique, CISH was performed as a primary amplification test if invasive tumor could not be separated from DCIS or if the tumor had heterogeneous protein expression by IHC (figure 1).

In the selected cases, CISH analysis was performed using the ZytoDot SPEC HER-2 Probe Kit (ZytoVision, Bremerhaven, Germany) or the FDA-approved SPoT-Light HER-2 CISH kit (Invitrogen, Carlsbad, CA, USA), according to the manufacturers’ instructions. A positive control was included in each CISH run and consisted of paraffin sections of a case known to be HER-2 amplified by CISH. Normal cells on the same slide, containing 2 copies, served as a “negative” control.

Per sample, nuclei of 30 randomly selected invasive breast cancer cells were counted. Samples with an average of < 4 spots/nucleus were considered unamplified and samples with > 6 spots/nucleus were considered amplified. If the average copy number was 4-6 spots/nucleus, nuclei of 30 additional cells were counted. An average of < 5 spots/nucleus was in these cases considered unamplified and an average of > 5 spots/nucleus was considered amplified [5]; [28].

The centromeric region of chromosome 17 (CEP-17) was used as an internal control. However, because polysomy 17 is believed to be a very rare event in breast cancer, and its clinical relevance is deemed questionable, we did not use the HER-2/CEP-17 ratio for result interpretation [29]; [30]. Moreover, in our experience, HER-2/CEP-17 ratio did not correlate as well with MLPA scores as the average HER-2 copy number did. We analyzed CEP-17 copy number gain in all 30 cases for which CEP-17 copy number was mentioned in the pathology report.

### Statistic analysis

The Chi-square test statistic was used to compare the frequencies of 0/1+, 2+ and 3+ IHC scores between the 4 laboratories. Furthermore, the frequencies of HER-2 gene amplification were compared between the 4 laboratories using Chi-square.

The concordance between protein expression determined by IHC and gene amplification assessed either by CISH, MLPA or both was assessed using the unweighted Cohen’s Kappa (K), not taking the IHC equivocal cases into account. A K value of 0.00–0.20 indicates a slight agreement, 0.21–0.40 suggests a fair agreement, 0.41–0.60 suggests a moderate agreement, 0.61–0.80 implies a substantial agreement, and finally a K value 0.81–1 indicates a perfect agreement. P-values < 0.05 were considered statistically significant. All reported p-values are 2-sided.

## Results

### MLPA validation study with CISH and IHC

We observed 100% concordance between MLPA and CISH in our validation study, if both methods were assessable and conclusive (81.8% of cases) (table 1). Concordance between MLPA/CISH and IHC was 94.1% (64/68) with a K value of 0.95 (95% CI 0.84–1.00), not taking the IHC equivocal cases into account (table 2).

**Table 1:** Concordance between MLPA and CISH in the validation study on 99 invasive breast cancer cases.

	CISH			
MLPA	No amplification	Amplification	na	Total
**No amplification**	66 (98.5%)	-	1 (1.5%)	67 (67.7%)
**Amplification**	-	15 (100%)	-	15 (15.1%)
**na/equivocal**	11 (64.7%)	-	6 (35.3%)	17 (17.2%)
**Total**	77 (77.8%)	15 (15.1%)	7 (7.1%)	99

**Table 2:** Concordance between MLPA/CISH and IHC in the validation study on 99 invasive breast cancer cases.

	MLPA/CISH			
IHC	No amplification	Amplification	na	Total
**Negative (0**–**1+)**	53 (94.6) a	-	3 (5.4)	56 (56.6)
**Equivocal (2+)**	24 (77.4) b	4 (12.9)	3 (9.7)	31 (31.3)
**Positive (3+)**	1 (8.3)	11 (91.7)	-	12 (12.1)
**Total**	78 (78.8%)	15 (15.1%)	6 (6.1%)	99

Abbreviations: na, not assessable.

a 6/53 (11.3%) lacked amplification by CISH following an equivocal or na MLPA result.

b 5/24 (20.8%) lacked amplification by CISH following an equivocal or na MLPA result.

### Interlaboratory differences in IHC scores

Table 3 illustrates the percentages of cases scored as 0/1+, 2+ or 3+ by IHC in the 4 pathology laboratories. The percentages of cases scored as 0/1+ and 2+ varied significantly between the laboratories (p < 0.0001 for both scores). On the other hand, the percentages of cases scored as positive (3+) were similar (p  =  0.974).

**Table 3:** Comparison of HER-2 IHC scoring percentages between 4 Dutch pathology laboratories.

Laboratory	A (n = 345)	B (n = 255)	C (n = 122)	D (n = 215)	Total (n = 937)
Negative (0–1+)	200 (58.0%)	183 (71.7%)	87 (71.3%)	168 (78.1%)	638 (68.1%) *
Equivocal (2+)	106 (30.7%)	42 (16.5%)	21 (17.2%)	20 (9.3%)	189 (20.2%) *
Positive (3+)	39 (11.3%)	30 (11.8%)	14 (11.5%)	27 (12.6%)	110 (11.7%)

* statistically significant (p < 0.05).

### MLPA and CISH for HER-2 amplification

**Figure 2:**
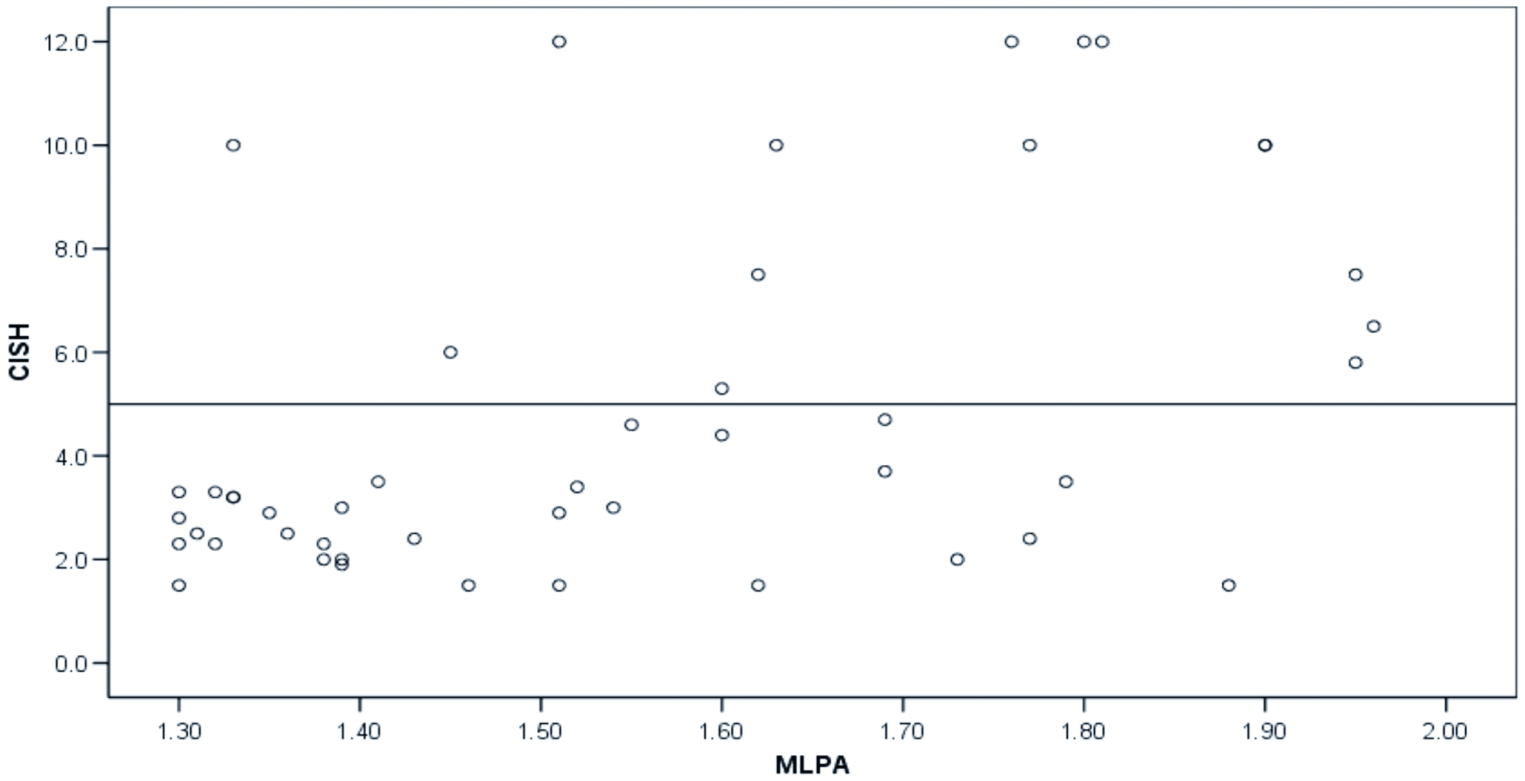
CISH and MLPA scores of MLPA equivocal cases. Scatterplot demonstrating MLPA and reflex CISH scores of 47/49 MLPA equivocal cases. Exact CISH copy numbers of 2 cases could not be retrieved. Reflex CISH demonstrated amplification in 15/49 (30.6%) equivocal cases.

The gene amplification test protocol presented in this study, comprising MLPA (and CISH in selected cases), gave 98.6% (773/784) conclusive results. First-line CISH was performed in 26 cases and was conclusive in 25 cases (96.2%). MLPA was used as a primary test to determine HER-2 gene amplification status in 758 cases and resulted in a conclusive and unequivocal result in 671 cases (88.5%). In the remaining 87 cases (11.5%) MLPA was either not assessable or equivocal. In 81/87 of these cases reflex CISH was performed, and in the remainder CISH could not be performed due to an insufficient amount of tumor tissue. CISH gave conclusive results in 77/81 cases (95.1%). In the remaining 4 cases CISH was not assessable due to poor tissue quality by bone decalcification or poor fixation. Of the 49 cases that had an equivocal MLPA result, reflex CISH demonstrated no amplification in 34 cases (69.4%) and amplification in 15 cases (30.6%) (figure 2).

We reviewed 30 cases for which the CEP-17 copy number was included in the pathology report. Three of them showed CEP-17 polysomy. In 1/3 use of the HER-2/CEP-17 ratio would have resulted in no amplification, whereas both HER-2 copy number and MLPA copy number ratio showed amplification.

### Concordance between IHC and MLPA/CISH

The concordance rates between IHC and MLPA/CISH were determined for a subset of 784/937 cases (83.7%) of which HER-2 gene amplification status was determined (table 4). Of the 490 IHC negative cases, 478 (97.6%) were not amplified, 4 (0.8%) presented with amplification, and 8 (1.6%) were not assessable by either MLPA or CISH. Of the 189 IHC equivocal cases, 171 (90.5%) were not amplified, 17 (9.0%) were amplified, and 1 (0.5%) was not assessable. Of the 105 IHC positive cases, 7 (6.7%) failed to demonstrate amplification, 97 (92.3%) were amplified, and 1 case (1.0%) was not assessable. Eleven cases (1.9%) in total demonstrated discordances between IHC and MLPA/CISH. The overall concordance between IHC and MLPA/CISH was 98.1% (575/586) with a K value of 0.94 (95% CI 0.90–0.97), not taking the IHC 2+ cases into account.

**Table 4:** Concordance between HER-2 protein expression determined by IHC and amplification determined by MLPA and/or CISH.

	MLPA/CISH (n = 784)			
IHC	No amplification	Amplification	na	Total
**Negative (0**–**1+)**	478 (97.6%)	4 (0.8%)	8 (1.6%)	490
**Equivocal (2+)**	171 (90.5%)	17 (9.0%)	1 (0.5%)	189
**Positive (3+)**	7 (6.7%)	97 (92.3%)	1 (1.0%)	105
**Total**	**656 (83.7%)**	**118 (15.1%)**	**10 (1.2%)**	**784**

Abbreviations: na, not assessable.

In total, 15.1% (118/784) of the cases tested revealed HER-2 gene amplification and were thus eligible for trastuzumab. The observed percentages of cases presenting with gene amplification did not vary significantly between the 4 laboratories (13.3%, 19.2%, 15.5% and 13.5% in hospitals A, B, C and D respectively; p = 0.287).

Trastuzumab would have been considered in 13.5% (127/937) of the patients if the 2007 ASCO/CAP guidelines [5] were applied. These 127 patients comprised 110 patients with protein overexpression at the 3+ level (merely 105 of these were co-tested by MLPA/CISH) and 17 cases with IHC overexpression at the 2+ level with confirmed gene amplification.

### Review of IHC/amplification discordant cases

**Figure 3:**
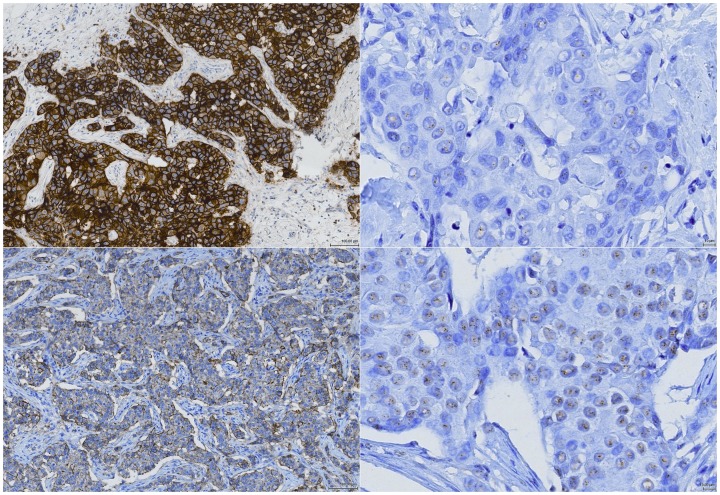
Representative images of 2 discordant cases (B2 and A1). Upper left: IHC of case B2 scored as 3+. Upper right: no amplification with CISH in case B2. Lower left: IHC of case A1 scored as 1+. Lower right: amplification with CISH in case A1. IHC images: 10x magnification. CISH images: 40x magnification.

The IHC sections of the 11 discordant cases were retrospectively reviewed by a breast pathologist (HS) and the CISH sections by a molecular technician (AH) to ensure or deny real discordance between IHC and MLPA/CISH (table 5). In 2/4 (50%) of the amplified cases originally scored as IHC 0/1+, review of the IHC score led to an adjustment to 2+ or 3+. Also, in 5/7 (71%) of the non-amplified cases originally scored as IHC 3+, IHC score was altered to 2+ or 1+ on review. CISH score was not adjusted in any of the initially discordant cases. In 4/11 discordant cases (D2, A1, B2 and B3) results remained discordant after retrospective review. As a final confirmation of the 4 discordant cases, we repeated IHC staining at a single location (laboratory A). Review of these IHC sections by a breast pathologist (HS) eventually led to the adaption of one more non-amplified 3+ case into a 2+ score (case B3), leaving 3 true discordant cases. Representative images of discordant cases A1 and B2 are presented in figure 3.

**Table 5:** Retrospective review of 11 discordant cases.

	IHC	MLPA (copy number ratio)	CISH (copy number)	Review IHC	Review CISH	Repeated IHC staining
D2 a	1+	A (2.47)	nt	0	na	0 b
A1 a	1+	Eq (1.60)	A (5.3)	1+	A (5.4)	1+ b
D1	1+	A (3.55)	A (>10.0)	2+	A (>10.0)	
B1	1+	A (2.31)	nt	3+	A (12.0)	
D4	3+	Eq (1.62)	NA (1.0–2.0, hg 7.0–8.0 (∼20%))	1+, hg 2+	NA (2.8)	
A3	3+	NA (1.24)	NA (3.5)	2+	NA (3.0)	
D3	3+	Eq (1.79)	NA (3.5)	2+	NA (3.5)	
A2	3+	NA (1.08)	nt	2+	NA (2.7)	
C1	3+	NA (0.99)	nt	2+	NA (2.0)	
B2 a	3+	Eq (1.77)	NA (2.4)	3+	NA (2.4)	3+ b
B3 a	3+	Eq (1.52)	NA (3.4)	3+	NA (4.98)	2+

Abbreviations: A, amplification; Eq, Equivocal; hg, heterogeneous; NA, no amplification; na, not assessable; nt, not tested.

a Amplification status would not have been determined properly when applying the ASCO/CAP protocol.

b True discordant cases.

## Discussion

Although IHC is the most commonly used first-line test for examining HER-2 status, HER-2 assessment by IHC can be problematic. First, IHC scores vary depending on the antibodies used [9]. Second, IHC is sensitive to pre-analytical variation, including tissue fixation [31]. This problem may especially be troublesome in laboratories serving several hospitals (like Symbiant B.V.), with inevitably suboptimal or delayed fixation during tissue transportation. This problem is expected to be exacerbated by the ongoing centralization of pathology services. Finally, IHC scoring is subjective and thus shows inter- and intra-observer variability [6]–[8]. Gene amplification tests may be less sensitive to these sources of error. As a second line test in case of an equivocal IHC result, ISH is now commonly applied, but it is relatively laborious and expensive. The quantitative MLPA technique has proven to be a reliable, less expensive and high-throughput test, and can therefore be a cost-effective alternative to ISH [11]–[14].

Our study aimed to prospectively assess the added value of co-testing every invasive breast cancer for HER-2 using a combination of IHC and MLPA in routine pathology practice. We analyzed data from consecutive cohorts of 937 invasive breast cancers derived from 4 Dutch pathology laboratories.

Despite standardized IHC protocols and double-review, our data reveal significant inter-laboratory variation in the percentages of cases scored as IHC 0/1+ and 2+. This variation clearly shows the pre-analytical, tissue processing and interpretation problems associated with IHC. The percentages of IHC 3+ cases were, however, quite comparable between the laboratories. Among biopsies, high rates of false-positive IHC scores were reported in several publications [32]; [33]. In our cohorts, we observed a significantly higher percentage of IHC 3+ cases among the biopsies than among the resections (17.7% vs. 10.2%; p  =  0.004). No further effort was done to determine whether these 3+ cases in biopsies contained more false-positives compared to these in resections.

MLPA as a single test produced conclusive results in 88.5% of cases. Our combined MLPA/CISH strategy, where the result of reflex CISH determined the definite amplification status, produced conclusive results in 98.6% of cases. Reflex CISH failed in 10/87 (11.5%) of cases, because of insufficient tumor material, bone decalcification or poor tissue fixation. Reflex CISH revealed an absence of amplification in 69.4% of the cases with equivocal MLPA results, and it was thereby a useful addition to MLPA, especially in the borderline cases.

We did not use the HER-2/CEP-17 ratio for CISH result interpretation. Several studies state that polysomy 17 is a very rare event in breast cancer, and its clinical relevance is deemed questionable [29]; [30]. Moelans *et al* showed that CEP-17 copy number was unrelated to the gains and losses of 17 genes located on chromosome 17, as determined by MLPA [30]. In our 3 cohorts from Symbiant B.V., only 3/30 cases showed CEP-17 polysomy, and in 1 case use of the HER-2/CEP-17 ratio would have resulted in no amplification, whereas both HER-2 copy number and MLPA copy number ratio showed amplification. Unfortunately, the CEP-17 copy number was counted and reported in 30 cases only.

The percentage of HER-2 amplified cases in our study (15.1%) was similar to that in previous studies [12]; [34]. Choritz *et al* [34] demonstrated an average of 14.6% (ranging from 7.6% to 31.6%), calculated with the results from 42 pathology laboratories. Moelans *et al* [12] detected HER-2 amplification in 14% of 518 invasive breast cancers. Moreover, in our study, the percentages of HER-2 amplified cases were similar between the 4 laboratories.

The 2007 ASCO/CAP guidelines [5] state that at most 5% of cases without protein overexpression should present with HER-2 gene amplification, and the percentage of cases with highly overexpressed protein lacking HER-2 gene amplification should not exceed 10%. The data presented in our study are in compliance with these guidelines, as 0.8% of cases scored as IHC 0/1+ revealed HER-2 gene amplification, whereas 6.7% of cases scored as IHC 3+ lacked gene amplification. Review of the 11 discordant cases revealed true discordance in only 3 cases, while in the remainder the original IHC score was incorrect or adapted after repeated IHC staining. Discordance between protein expression and gene amplification described by others ranged from 0% to 11.5% IHC negative/amplified tumors and from 0% to 21.9% IHC positive/non-amplified tumors [7]; [10]–[12]; [15]–[18].

The central question in view of the discrepancies between IHC and gene amplification tests is whether IHC 3+ patients without HER-2 gene amplification and IHC 0/1+ patients with gene amplification respond to trastuzumab. Thus far, there is no consensus on the gold standard for HER-2 testing, because no technique is perfectly able to identify patients likely to benefit from trastuzumab therapy [5]. Although there are some indications that gene amplification may be a better predictor for response to trastuzumab than protein overexpression [35]–[39], there are also indications that amplified tumors particularly respond to trastuzumab if they present with protein overexpression at the 3+ level [37]; [40]; [41]. The final answer to this question could probably only be provided by a meta-analysis combining response data for such IHC/amplification discrepant patients from different clinical trastuzumab trials, but so far this has not been executed. Such an analysis is eagerly awaited, in view of the high costs of trastuzumab and its potential side effects.

The question whether laboratories should switch to the use of a first line gene amplification test for all cases is subject of an ongoing debate. Some laboratories already perform frontline ISH testing [42]; [43]. In Australia, an HER-2 ISH test is required for all early breast cancer patients [44], and patients demonstrating HER-2 gene amplification are eligible for trastuzumab, regardless of their IHC score. The decision to stratify patients according to ISH results was based on guidelines [39] preferring ISH over IHC because of its greater test accuracy, objectivity and reproducibility.

Performing a reflex gene amplification test routinely in all patients has several advantages. First, gene amplification testing seems to be less sensitive to pre-analytical factors, is more quantitative and easier to interpret, and may therefore reduce variation between labs. Second, it may serve as quality control for IHC scoring as illustrated in our study, where IHC score was, on review, adjusted in over half of the discrepant cases after gene amplification testing. Third, it will reveal gene amplified cases among the IHC 0/1+ patients that will likely respond to trastuzumab, and who would possibly be denied an effective therapy based on IHC alone. Fourth, it may identify IHC 3+ patients without gene amplification, who may respond less well to trastuzumab, although further evidence is required here as discussed above.

In order to control costs of HER-2 co-testing, a low-cost high-throughput gene amplification test like MLPA is demanded. MLPA reagent costs are approximately 30% lower than CISH reagent costs. Although the hands-on time to perform the tests is similar for MLPA and CISH, MLPA analysis time is approximately 5 times shorter than CISH analysis time. Furthermore, in contrast to CISH, which needs to be double-reviewed by a technician and a pathologist, MLPA produces a directly interpretable quantitative value and does not involve analysis by a pathologist. A qualified pathologist should, however, mark areas of invasive breast cancer to be included in the MLPA analysis. MLPA is thereby particularly cost-effective for the analysis of large sample sizes and is accordingly an ideal technique for HER-2 co-testing.

In conclusion, assessment of HER-2 gene amplification by MLPA is a low-cost, quantitative and high-throughput technique with near perfect concordance with CISH. When routinely applied in all breast cancer patients, it may improve the quality of HER-2 testing by reducing variation in HER-2 testing between labs, serving as a quality control for IHC, identifying gene amplified cases among the IHC 0/1+ patients that will likely respond to trastuzumab as well as IHC 3+ cases without gene amplification that may respond less well to trastuzumab.
